# Influenza Vaccination for the Prevention of Cardiovascular Disease in the Americas: Consensus document of the Inter-American Society of Cardiology and the Word Heart Federation

**DOI:** 10.5334/gh.1069

**Published:** 2021-08-05

**Authors:** Álvaro Sosa Liprandi, María Inés Sosa Liprandi, Ezequiel José Zaidel, Gabriel M. Aisenberg, Adrián Baranchuk, Eduardo Costa Duarte Barbosa, Gabriela Borrayo Sánchez, Bryce Alexander, Fernando Tomás Lanas Zanetti, Ricardo López Santi, Ana Girleza Múnera-Echeverri, Pablo Perel, Daniel Piskorz, Carlos Enrique Ruiz-Mori, Jorge Saucedo, Osiris Valdez, José Ramón González Juanatey, Daniel José Piñeiro, Fausto J. Pinto, Fernando Stuardo Wyss Quintana

**Affiliations:** 1 School of Medicine, University of Buenos Aires, AR; 2 Cardiology Department, Sanatorio Güemes, Buenos Aires, AR; 3 InterAmerican Society of Cardiology, AR; 4 Pharmacology Department, School of Medicine, University of Buenos Aires, AR; 5 University of Texas John P and Kathrine G McGovern School of Medicine, Houston, Texas, US; 6 Division of Cardiology, Kingston Health Science Center, Queen’s University, Kingston, Ontario, CA; 7 Cardiology Department, Hospital Sao Francisco-Santa Casa, Porto Alegre, BR; 8 Artery LatAm, LatinAmerican Society of Hypertension, BR; 9 Cardiology Department, Mexican Social Security Institute, Mexican National Association of Cardiologists, MX; 10 Cardiology Department, CIGES, Universidad de La Frontera, Temuco, CL; 11 Cardiology Department, Hospital Italiano de La Plata, Buenos Aires, AR; 12 Argentine Federation of Cardiology, AR; 13 Cardiology Department, Hospital General Medellin Luz Castro de Gutierrez E.S.E. Medellin, CO; 14 Department of Non-communicable Disease Epidemiology, London School of Hygiene and Tropical Medicine, GB; 15 World Heart Federation, Geneva, CH; 16 Cardiology Department, British Hospital of Rosario, Santa Fe, AR; 17 Cardiology Department, INEN, Lima, PE; 18 Cardiology Department, Froedtert Hospital and Medical College, Milwaukee, US; 19 Cardiology Department, Centro Médico Central Romana, La Romana, DO; 20 Central America Society of Hypertension, DO; 21 Cardiology Department, Hospital Clínico Universitario de Santiago de Compostela, Spanish Society of Cardiology, ES; 22 Universidad de Buenos Aires, AR; 23 Cardiology Department, Hospital Santa María, PT; 24 University of Lisbon, PT; 25 Cardiosolutions Guatemala, GT

**Keywords:** influenza, influenza vaccination, cardiovascular disease, myocardial infarction, consensus

## Abstract

**Background:**

Cardiovascular mortality is decreasing but remains the leading cause of death world-wide. Respiratory infections such as influenza significantly contribute to morbidity and mortality in patients with cardiovascular disease. Despite of proven benefits, influenza vaccination is not fully implemented, especially in Latin America.

**Objective:**

The aim was to develop a regional consensus with recommendations regarding influenza vaccination and cardiovascular disease.

**Methods:**

A multidisciplinary team composed by experts in the management and prevention of cardiovascular disease from the Americas, convened by the Inter-American Society of Cardiology (IASC) and the World Heart Federation (WHF), participated in the process and the formulation of statements. The modified RAND/UCLA methodology was used. This document was supported by a grant from the WHF.

**Results:**

An extensive literature search was divided into seven questions, and a total of 23 conclusions and 29 recommendations were achieved. There was no disagreement among experts in the conclusions or recommendations.

**Conclusions:**

There is a strong correlation between influenza and cardiovascular events. Influenza vaccination is not only safe and a proven strategy to reduce cardiovascular events, but it is also cost saving. We found several barriers for its global implementation and potential strategies to overcome them.

## Introduction

Cardiovascular (CV) mortality continues to be the main cause of death in developed countries as well as in emerging economies. Respiratory infections, particularly those caused by the influenza virus, contribute significantly to morbidity and mortality throughout the world, representing the third cause of mortality in several Latin American nations, particularly in low- to middle-income countries [1]. Analysis of the evidence based on systematic reviews and meta-analysis of epidemiological studies show a consistent association between respiratory infections and the incidence of acute myocardial infarction (AMI) and CV mortality [2,3,4,5,6,7,8,9,10,11].

Numerous deaths and CV complications take place during flu epidemics, especially in vulnerable populations. Patients with chronic CV diseases are particularly at risk during this period and represent a population that could be targeted for vaccination, as one of the main objectives in public health today is to reduce the impact of CV disease in the general population. Current COVID-19 pandemic has reflected the strong link between respiratory infections and CV diseases, both as risk group and as trigger for new events, and that reinforced the purpose of this consensus.

In the last 15 years, influenza vaccination (IV) in high-risk populations has become an effective strategy to reduce the incidence of respiratory infections and therefore associated CV complications. However, the prescription of the IV is not common in routine cardiology practice, and vaccination rates vary widely among high-risk vulnerable populations in different regions of the world [12]. This reluctance of cardiologists to incorporate immunization as a routine CV prevention strategy for their patients has been observed in Latin American countries [13,14].

The incomplete application of existing recommendations prompted us to perform an in-depth analysis of the literature regarding IV and CV events and the possible barriers to its implementation. Within this framework, the Inter-American Society of Cardiology (SIAC) and the World Heart Federation (WHF) decided to promote the development of a consensus on the role of influenza immunization as a cardiovascular prevention strategy.

The purpose of the document was to critically analyze the contemporary evidence that supports the use of the IV in adults in order to reduce the rate of CV events and its effect on the burden of the disease in our region, as well as to analyze the difficulties and barriers in its implementation. This was done based on the evidence available in the literature and the experience of the participants in clinical practice.

## Methods

The consensus document was made using the modified RAND/UCLA methodology. This method is based on the available scientific evidence and the collective judgment and clinical experience of a panel of experts. It is a combination of the Delphi technique with that of nominal groups [15,16].

A multidisciplinary team composed of experts in the management and prevention of CV disease from the Americas was convened by the IASC and participated in the process and the formulation of statements. A coordinating committee of two experts and one scientific secretary was gathered, along with a recommendation-formulating group that included the coordinating committee, fourteen more experts, and two consultants who provided the final review of the document. A content index and a list of seven relevant clinical questions were developed during the kickoff meeting (association between influenza and CV events, efficacy of IV in different risk groups, safety of the vaccination and different vaccination schemes, cost-effectiveness, implementation barriers, and proposals for increasing vaccination rates). A nonsystematic expert search of the available publications related to these clinically relevant questions was conducted in May/Jun 2020 in databases including MEDLINE via PubMed, CENTRAL (Cochrane), Scielo and LILACS. Priority was given to those that were relevant to the Americas or conducted in American countries. A total of 245 publications were retrieved. Question 5 required a systematic review of publications in four databases. To evaluate cost-effectiveness (CE) and the quality of the publications, the list of verifications of the international research society in pharmacoeconomics ISPOR 200 was used. The review was carried out from June to September 2020, on the databases MedLine (via Pubmed, n = 124) DARE, HTA, and EED (via CRD-York, n = 51). Finally, 45 CE studies of influenza vaccination remained for analysis. An extended version of this document, including the full methodology of the systematic review and the results of each article is freely available at IASC website, www.siacardio.com [17].

Statements were debated during a structured virtual meeting. A total of 38 statements and conclusions were included. Statements that achieved unanimity (100% agreement) or consensus (80% agreement) were accepted. Statements were formally categorized with their level of evidence and degree of recommendation according to AHA/ACC Guidelines (Table 1) [18].

**Table 1:** Class of Recommendation and Level of Evidence, ACC/AHA criteria [19].

Class of Recommendation (CoR)		

I	Strong	Benefit >>> Risk
IIa	Moderate	Benefit >> Risk
IIb	Weak	Benefit > Risk
III	No benefit or harm	Benefit = Risk, or Risk > Benefit
**Level of Evidence (LoE)**		

A		High quality evidence of >1 RCT or meta-analysis of them
B-R	Randomized	Moderate evidence from 1 RCT
B-NR	Non-Randomized	Moderate evidence from well-designed nonrandomized trial
C-LD	Limited Data	Randomized or nonrandomized clinical studies of limited quality. Meta-analysis of those studies. Mechanistic studies.
C-EO	Expert Opinion	Consensus of experts opinion based on clinical experience

This document was supported by an unrestricted grant to the Inter-American Society of Cardiology provided by Sanofi Pasteur to the World Heart Federation. Funder had no role in the drafting, evidence review, meetings, or publication process.

## Results

### Association between respiratory infections and cardiovascular events

A recent systematic review reported a consistent association between influenza and AMI, as well as weak evidence of an association with CV death, heart failure (HF), and stroke [19]. Likewise, influenza epidemics are associated with an increase in autopsy-confirmed coronary death [20]. Acute respiratory infections have been considered a trigger for AMI and CV mortality, attributable to a possible inflammatory and prothrombotic effect. Recent respiratory symptoms are associated with the development of AMI (OR 2.1, 95% CI, 1.4–3.2) [5]. Additionally, in patients with laboratory-confirmed influenza infection, the incidence rate of AMI and stroke was six and eight times higher, respectively, during the seven days after infection, compared with control intervals [21,22]. Influenza as a trigger for CV events meets the Bradford Hill causality criteria (strength of association, biological gradient, temporality, consistency, seasonality, coherence, analogy, and biological plausibility) [23].

Influenza has also been found to be associated with arrhythmias and HF hospitalizations. In 11,374 patients with influenza, an 18% increase in atrial fibrillation (AF) was observed [24], as well as a 24% increase in hospitalization rates for HF (incidence rate, 1.24; 95% CI, 1.11–1.38; p < 0.001) [25]. In an analysis of more than 8 million HF hospitalizations, those with concomitant influenza infection had higher rates of mortality and respiratory and renal failure [26].

The association between influenza and venous thromboembolic events (VTE) is less clear, with the information in the literature showing conflicting results [27,28,29,30,31]. The current state of knowledge in this domain is additionally complicated by further conflicting results regarding the CV impact of influenza virus types and subtypes in VTE [32,33,34]. As pathophysiological hypothesis, cytopathic effects (invasion of endothelial cells), indirect effects mediated by the host’s immune response, virus invasion in atheromatous plaques, infiltration of inflammatory cells, and increased cytokines, with consequent activation of the coagulation cascade, have been described [35,36,37]. A recently developed model supports the association between inflammation and arterial thrombosis as a fundamental link in the relationship between influenza and CV mortality [38]. Increased quantity and quality of scientific evidence is required to support this association, since current knowledge is based mainly on retrospective cohorts. Results of prospective clinical trials under development are anticipated and will add substantially to the current body of knowledge [39].

The conclusions related to this issue are summarized in Table 2.

**Table 2:** Association between respiratory infections and cardiovascular events.

	Conclusions	CoR	LoE

A	Influenza-like respiratory infections are associated with CV events during follow-up (AMI, stroke, hospitalizations for HF, AF, and CV death).	I	B-NR
B	The association between influenza and thromboembolic disease is controversial.	IIb	C-LD
C	There is not strong enough evidence to determine the incidence or mortality from CV disease for different types and subtypes of influenza viruses.	IIb	C-LD
D	There are proven pathophysiological mechanisms that explain the association of influenza with cardiovascular events.	IIb	C-LD

### Clinical efficacy and/or effectiveness of influenza vaccines in cardiovascular events reduction

#### Coronary artery disease

Influenza vaccination may be an effective strategy to reduce CV events in patients with pre-existing CV disease. Randomized clinical trials (RCTs) have had dissimilar results regarding cardiovascular death and major cardiovascular events (MACE). However, results of two meta-analyses have shown that influenza vaccine reduced both CV death and MACE, compared to the control group, by approximately 50%.

In the systematic review by LeBras et al. [40], two meta-analyses including the same RCTs were compared [41,42,43,44]. The meta-analysis by Udell et al. compared IV versus placebo or standard treatment in four studies in which 1655 patients had pre-existing CV disease [45]. The primary endpoint was extended MACE, defined as CV death or hospitalization for AMI, unstable angina, stroke, HF, or emergency coronary revascularization. In the subgroup analysis with pre-specified CV disease, IV significantly reduced both MACE (RR 0.57, 95% CI 0.41–0.79, I2 = 14%) and CV death (RR 0.50, 95% CI 0.27–0.95, I2 = 15%). In patients with recent acute coronary syndrome (≤1 year) (n = 815), IV significantly reduced MACE (RR 0.46, 95% CI 0.33–0.64, I2 = 0%) but not CV death (RR 0.44; 95% CI 0.17–1.15; I2 = 38%) or all-cause mortality. The findings are based on a relatively small number of CV events (246 MACE and 97 CV deaths) and arise from trials that varied in study design, expected primary outcomes, and patient populations. There was no difference in MACE or CV death in the subgroup of patients with stable coronary artery disease (n = 840).

A subsequent Cochrane Collaboration meta-analysis of eight RCTs included the same studies, but with a total of 1682 patients with established CV disease [46]. Similar to reported data in the Udell meta-analysis, CV death was significantly lower with IV (RR 0.44, 95% CI 0.26–0.76, I2 = 0%). However, there was no difference in CV death in the subgroup of patients with acute coronary syndrome (n = 350) (RR 0.46, 95% CI 0.04–5.20, I2 = 58%) or stable angina and elective coronary angioplasty (n = 602) (RR 0.35, 95% CI 0.07–1.73, I2 = 0%).

A recent meta-analysis of four RCTs and 12 observational studies demonstrated that IV was associated with a 25% and 18% RR reduction in all-cause mortality and CV mortality, respectively, in patients with previous CV disease. The reduction in mortality was probably driven in part by a 13% RR reduction in MACE [47].

#### Heart failure

Observational studies have suggested that IV could reduce events during follow-up in patients with HF [12,48]. Mohseni et al. examined the association between IV and the risk of hospitalization in patients with HF, through a primary care database associated with the registry of hospitalizations in England during the years 1990–2013. In the 52,202 patients analyzed, IV was associated with a lower risk of CV hospitalization (HR = 0.73, 95% CI 0.71–0.76) and all cause hospitalization (HR = 0.90, 95% CI 0.95–0.98) [49]. The effect was somewhat greater in younger patients, without gender differences.

Modin et al., using data from the Danish national patient registry, analyzed the relationship between influenza vaccination and survival in patients with a recent diagnosis of HF, between the years 2003–2015. After adjusting the results for comorbidities, medication, income, and educational level, influenza vaccination was associated with an 18% reduction in total mortality (HR = 0.82, 95% CI 0.81–0.84, p < 0.001) and cardiovascular mortality (HR = 0.82, 95% CI 0.81–0.84, p < 0.001). Annual, timely, and sustained vaccination over the years was associated with a greater risk reduction of death when compared to intermittent vaccination [50].

Rodrigues et al. recently conducted a systematic review and meta-analysis of the HF clinical trials available to date. Six cohort studies with 179,158 patients were included in the analysis. IV was associated with a lower risk of all-cause mortality (HR = 0.83, 95% CI 0.76–0.91, I2 = 75%) and a significant reduction in HF hospitalizations (HR = 0.69, 95% CI 0.55–0.86). No significant effect was observed on CV mortality (HR = 0.92, 95% CI 0.73–1.15) and all-cause hospitalization (HR = 1.01, 95% CI 0.92–1.11). The level of certainty of the evidence was considered low due to the quality of the data obtained in the studies [51].

In conclusion, although there is proven evidence of the benefit of IV in the context of patients with pre-existing CV disease, given the limitations of the data due to the small sample size, low event rate, potential confounders and bias, results of prospective and randomized studies of greater power are required to allow definitive conclusions [39,52].

#### Primary prevention

Patients with hypertension (HTN), diabetes, and AF have an increased risk of complications associated with influenza. The mostly observational studies done to date have shown a protective effect of IV for these three populations, but due to the limitations of the studies, it is not possible to establish strong recommendations [53,54,55].

Although influenza is a universal disease and affects all age groups, 90% of deaths and 50% of hospitalizations are among people over 65 years of age. For this reason, historically vaccination recommendations had been directed at people above that age and those with risk factors (chronic diseases, immunosuppressed, etc.) [56].

The prospective cohort studies carried out by Nichol et al., in health organizations in the United States for patients older than 65 years deserve special mention. In the 1999–2000 epidemic season, the effectiveness of the vaccine was 29% in reducing hospitalizations for influenza-pneumonia, 27% in hospitalizations for HF, 23% in hospital admissions for CV disease, and 24% in all-cause hospitalizations. The reduction in deaths from all-cause mortality was 36%. In those without established CV disease, diabetes, or associated comorbidities (low-risk groups), influenza vaccination was also associated with a significant reduction in CV events at follow-up (CV, cerebrovascular, and all-cause mortality and hospitalizations) [57,58,59,60].

The conclusions and recommendations related to this issue are summarized in Table 3.

**Table 3:** Clinical efficacy and/or effectiveness of influenza vaccines in cardiovascular events reduction.

	Conclusions	CoR	LoE

A	IV in patients with coronary artery disease is associated with a reduction in CV events.	I	B – R
B	In patients over 65 years of age at low risk (absence of comorbidities, without established CV disease or diabetes) IV is associated with a reduction in CV events.	I	B – NR
C	Annual IV in patients with HF is associated with a reduction in all-cause mortality and HF hospitalization.	IIa	B – NR
D	In patients with diabetes or hypertension, in the absence of established CV disease, the IV could be associated with a reduction in CV events.	IIb	C – LD
	**Recommendations**		

A	IV for patients with a recent acute coronary syndrome (≤1 year)	I	B – R
B	Annual IV for patients >65 years even in the absence of CV disease or risk factors.	I	B – NR
C	Annual IV for patients with chronic coronary artery disease with or without history of revascularization.	IIa	B – R
D	Annual IV for patients with HF.	IIa	B – NR
E	Annual IV for patients with diabetes or hypertension without established CV disease.	IIb	C – LD

### Safety of the influenza vaccine in patients with cardiovascular disease

The adverse events of IV can be divided into those attributed to the vaccine, its preservatives, inoculation, and the interaction with other drugs and vaccines. The Institute of Medicine (IOM) of the United States National Academy of Sciences found no evidence of causality between IV and a wide range of studied immunologic or neurologic events [61,62,63,64,65,66,67,68,69,70,71,72,73,74,75,76].

Currently available evidence suggests that the majority of people can be vaccinated, even with a documented allergy to eggs [77,78,79,80,81,82]. In cases of prior anaphylaxis, patients should be monitored for 30 minutes.

Presumed IV adverse events could represent manifestations of other winter respiratory pathogens as a natural disease [73,74].

Patients with CV diseases often require anticoagulants. There were no significant differences between the subcutaneous or intramuscular routes for administration of the influenza vaccine in the generation of hematomas, although there is a greater local reaction (pain and erythema at the puncture site on the first day) with the former [83]. Patients with higher INR (International Normalized Ratio) values do not present an increased risk of complications. There is no evidence that IV produces considerable changes in the INR of patients, and on the other hand, immunogenicity is similar between individuals who use oral anticoagulants or not [84]. Subdeltoid intramuscular administration produces a slight increase in the incidence of bursitis [85].

Regarding other CV drugs, no interactions have been found between influenza vaccines and antiplatelet agents, angiotensin converting enzyme inhibitors, angiotensin II receptor blockers, digitalis, amiodarone, flecainide, and diuretics [86]. The combination of aspirin and live attenuated influenza vaccine (LAIV) has been associated with the risk of Reye’s syndrome [87].

Vaccines for influenza and pneumococcus are mainly recommended in people over 65 years of age, or people with established CV disease given their susceptibility to both diseases [88]. The use of dual vaccination showed additive or synergistic preventive effects compared to separate administration or absence of vaccination, with adequate effectiveness and safety [89]. Concurrent use does not affect immunogenicity or safety, even in people with chronic respiratory diseases [90]. However, it increases the rate of mild local and systemic adverse events related to the injection site [90].

In the context of the COVID-19 pandemic, messenger RNA vaccines, vector vaccines (Adenovirus with Spike protein) and inactivated virus vaccines have been developed and approved to date. In relation to vaccination for influenza, the Argentine Respiratory Medicine Association recently suggested prioritizing vaccination for COVID-19 and waiting for an interval of at least 14 days between vaccines [91].

The formal contraindications are specific to each vaccine label and described elsewhere [92], the conclusions and recommendations related to this issue are summarized in Table 4.

**Table 4:** Safety of the influenza vaccine in patients with cardiovascular disease.

	Conclusions/Recommendations	CoR	LoE

A	The different flu vaccines are generally safe; the reduction in the incidence of epidemic influenza is significantly greater than the incidence of adverse effects.	I	A
B	Co-administration of injectable IV and warfarin is safe, requiring only longer pressure at the intramuscular injection site. There is insufficient information to support determining the INR before or at the time of administration of the vaccine.	II a III	B-R C-EO
C	Co-administration of influenza and pneumococcal vaccines is safe and immunogenic. Co-administration was associated with a higher rate of adverse events, albeit mild.	II a II b	B-R C-LD
D	In the context of COVID-19 pandemic, it may be beneficial the SARS-CoV-2 vaccination and then IV after an interval of at least 14 days.	IIb	C-EO
E	It is recommended not to administer the LAIV together with aspirin in children given the risk of Reye’s syndrome. The LAIV is generally not recommended for patients with CV disease.	III III	C-EO C-EO
F	Individuals with history of severe egg anaphylaxis may receive chicken embryo-based vaccine but should be monitored for at least 30 minutes after the administration.	IIa	B-R

### Efficacy of different immunization schedules

Both the efficacy in immunological terms and the clinical effectiveness of IV appear to decrease with age. This fact, linked to immune senescence, represents a state of dysregulation of immune function that predispose older patients to a greater susceptibility to infections of any type, in addition to other diseases that compromise the immune system, and the presence of comorbidities.

Although current IVs are immunogenic, mutations in the virus can reduce their effectiveness. Strategies focused on increasing immunogenicity or the spectrum of antiviral coverage include the use of high doses of vaccine, quadrivalent vaccines, adjuvanted vaccines, and those prepared in cell cultures. The elderly and immunocompromised are those who obtain the greatest benefit from these options [72,93,94,95,96,97,98,99,100,101,102,103,104,105,106,107].

The benefit of using higher than conventional doses in older patients has recently been demonstrated. A randomized, double-blind study conducted in the United States and Canada on 31,989 patients over 65 years of age has documented a 24% superior efficacy of high dose trivalent inactivated virus vaccine against lab-confirmed influenza, when compared to standard dose (60 μg vs 15 μg of hemagglutinin) [108]. A recent study reports that high dose vaccine is 14% more effective in reducing cardiovascular hospitalizations [109]. The increased protection of higher dose vaccine against various endpoints is also supported by a recent meta-analysis of data from 34 million older adults over 10 consecutive seasons [110]. The use of high doses induced significantly higher antibody responses and provided better protection against laboratory-confirmed influenza. Patients with a history of CV or chronic respiratory diseases who received high dose vaccine had a lower frequency of pneumonia or cardio-respiratory complications [110].

In a systematic review and meta-analysis of 7 trials in Canada, the authors concluded that in adults older than 65 years, high-dose IV was well tolerated, more immunogenic, and effective in preventing influenza infections than the standard-dose vaccine [111]. However, more pragmatic trials are needed to determine whether higher efficacy translates into greater clinical effectiveness of the vaccine in this population.

Recently, Vardeny et al., in a randomized double-blind study that included 5,260 high-risk patients with CV disease, did not find significant differences in mortality and CV hospitalization when they compared high doses of trivalent vaccine and standard doses of quadrivalent vaccine [93]. Some criticism to that trial rose regarding baseline risk, number of patients, vaccine effectiveness, as well as type of outcomes analyzed [112,113].

Quadrivalent vaccines have a safety profile similar to that of the trivalent vaccine. In seasons with relatively high influenza B activity, the quadrivalent vaccine appeared more protective than the trivalent [114,115].

The deep subcutaneous/intramuscular route of administration may reduce morbidity and mortality from seasonal influenza. Higher antibody levels have been identified after boosting with the application of attenuated virus vaccines intradermally (DNA-IIV3) and intramuscularly (IIV3-IIV3), although with greater swelling and local redness, compared to other routes of administration; systemic reactogenicity was similar between regimens [116].

Intranasal application not only induces IgG antibodies, but also activates the secretory IgA antibodies of the respiratory tract epithelium (S-IgA), mimicking natural infection [117]. For this reason, some experts point out that the intranasal route could be a safe and effective strategy [118,119].

Finally, with regard to the time of vaccination, it is always recommended to use the vaccine prior to the onset of influenza circulation. If such seasonality is not observed in some of the tropical countries, some authors suggest that a fixed administration strategy every six months could be used [120].

The conclusions and recommendations related to this issue are summarized in Table 5.

**Table 5:** Efficacy of different vaccination schedules.

	Conclusions/Recommendations	CoR	LoE

A	The vaccine should be administered at least annually before the annual season in which the incidence of influenza increases, or at the beginning of the season.	I	A
B	The high-dose inactivated influenza vaccine (IIV3-HD) is recommended compared to the standard dose (IIV3-SD) because it is more immunogenic, effective, and because it reduces cardiorespiratory outcomes	IIa	B-R
C	The quadrivalent inactivated influenza vaccine is recommended compared to the trivalent because it offers a broader protection.	IIa	B-NR
D	Adjuvant vaccines are indicated in elderly patients, with suboptimal immune responses, or when rapid responses to smaller doses are required during a pandemic.	IIa	B-NR
E	Influenza vaccines developed integrally in cell culture are more immunogenic than those developed in chicken embryos, requiring lower doses, and maintaining a comparable biosafety profile.	IIa	B-NR
F	The benefit regarding CV outcomes between IIV3-HD and quadrivalent vaccine could not be established due to methodological limitations in the only randomized clinical trial.	IIb	B-R
G	In tropical countries where it is proven that there is no seasonal variation in influenza, biannual vaccination could be beneficial, although there are still no studies to support this recommendation.	IIb	C-EO

### Cost-effectiveness of influenza vaccination

Based on the systematic review carried out, 45 cost-effectiveness (CE) studies were identified, and the results were stratified according to the population analyzed [17].

- Over 65 years: In 23 of 25 studies, vaccination was considered cost-effective [121,122,123,124,125,126,127,128,129,130,131,132,133,134,135,136,137,138,139,140,141,142,143,144,145]. The studies were carried out with economic models in 20 countries (including eight countries in the Americas) and analyzed vaccination with trivalent vaccine, or CE of the use of high-dose trivalent, with adjuvants, or quadrivalent. The results were similar in both non-industry sponsored (n = 20) and sponsored (n = 5) studies. Only three of the 25 studies included CV outcomes in the cost analysis modeling. The outcomes in which CE was demonstrated were ambulatory cases of influenza-like illness (ILI), hospitalizations for pneumonia, hospitalizations in intensive care units, mortality, and quality-adjusted life years (QALYs).
- Age 50–64 years: In seven of the eight studies identified that specifically analyzed this age group [138,146,147,148,149,150,151,152], IV was cost-effective for outpatient or hospitalized ILI events, pneumonia, and death. Four of the studies included CV outcomes.
- Diabetes: Two studies that included only diabetic patients were identified. In both, IV was cost-effective to reduce hospitalizations for ILI (Turkey), or CV outcomes and hospitalizations (China) [153,154]
- Heart failure: We did not identify any CE studies of IV that have exclusively looked at these patients. However, it should be considered that the mean age of subjects with HF ranges between 70 and 80 years [155,156], so it is possible that this population is represented in CE studies of the vaccine in these age groups.
- Coronary artery disease: We identified three studies that analyzed subjects after ACS [157,158,159]. In the United States and South Korea, it was cost-effective. In Thailand, for patients after a heart attack, but under 50 years of age, the CE was borderline, while after 50 years it was cost-effective. The outcomes included in the models were hospitalization for ILI and MACE.
- Patients with chronic diseases (including CV disease, stroke, or diabetes, among others): We found seven publications [137,160,161,162,163,164,165], where the CE of IV was evaluated in subjects considered ‘at risk’, regardless of age. This series of studies were conducted in high-income countries, all sponsored, and in them; quadrivalent vaccine was cost-effective to reduce cases of outpatient or hospitalized ILI.

Although our aim was to evaluate the CE of individual vaccination in risk groups, another approach as a preventive measure is mass vaccination, which was shown to be cost-effective in a model carried out in the United States [166,167]. Compared with other primary prevention strategies such as breast or colon cancer screening, or control of arterial hypertension, the CE of vaccination is of a similar magnitude [168,169].

In the Americas, we identified CE studies with positive results in eight countries: Canada, United States, Mexico, Costa Rica, Panama, Colombia, Brazil, Argentina and in tropical countries where there are seasonal peaks of influenza [124,126].

With regard to the types of vaccines, the information about disease costs and formal CE analysis in low- and middle-income countries (like most in the Americas) is of low quality or totally absent for some of the parameters, and therefore, for certain authors [170], it may not be a priority to use more complex vaccines than the conventional trivalent, from an economic point of view. Recently, after our systematic review and during the editorial process, a review was published including economic evaluation of high dose IV for people older than 65 years from USA and Canada. In that review, high-dose IV was cost-effective and cost saving, pulled by the economic benefit of CV events reduction [171].

The conclusions related to this issue are summarized in Table 6.

**Table 6:** Cost-effectiveness of influenza vaccination.

	Conclusions	CoR	LoE

A	Vaccination for influenza with a trivalent vaccine is a CE strategy: In adults in general, it is CE for the reduction of ambulatory ILI cases, hospitalizations for pneumonia, quality-adjusted life years and total mortality.	I	B-NR
B	Vaccination for influenza is CE when evaluating CV outcomes: From an economic point of view, vaccination is reasonable in those over 50 years of age, and regardless of age in subjects with diabetes, coronary artery disease, or other established CV diseases. In the United States, mass vaccination was CE compared to vaccination only to risk groups.	IIa	B-NR
C	Vaccination for influenza is as CE as other primary health prevention strategies (colon cancer screening, breast cancer screening, or control of arterial hypertension). Vaccination for influenza is CE in tropical countries.	IIa	C-LD
D	There is little to no information on CE of influenza vaccines with new technologies (with adjuvants, tetravalent, high doses) in low-resource countries, so there is not enough evidence yet to recommend one over the other, from the pharmacoeconomic point of view specifically.	IIb	C-EO

### Barriers to influenza vaccination implementation related to physicians, patients, and their context

Despite the benefits related to IV and the recommendations for its prescription by scientific societies and health regulatory agencies, vaccination rates globally, as well as in the Americas, are lower than desired [172]. This fact is explained by the presence of implementation barriers that involve doctors, patients, and health systems. An adequate diagnosis and recognition of each barrier is essential to generate potential strategies that allow increasing vaccination rates.

As with other prevention measures, medical knowledge through continuous education, clear regulations, and conviction regarding the risk-benefit ratio seem to be the main determinants of the implementation of an intervention.

The personal experience of the physician, as well as that of other health workers with influenza immunization, also appears to be a determining factor in future recommendations for patients. When ‘missed opportunities’ were analyzed in unvaccinated patients, lack of recommendation during medical visits was identified as the main cause. Seen in another way, when doctors have a positive attitude and recommend the vaccine, the immunization rate increases considerably, generating an effective vaccination between 50% and 93% of cases in different series [173,174,175,176,177,178,179,180,181,182,183]. A full list of the results of the analyzed studies is available in the large version of this consensus [17].

Specialist physicians may be reluctant to carry out primary prevention interventions [183]. Another great limitation in effective vaccination involves complex behavioral attributes related to the psychological aspects of patients [184,185,186]. There are approximately 500 articles that analyze behavioral aspects that determine vacillation in the vaccination decision. These aspects are grouped into complacency (example: low perceived risk of becoming ill or presenting serious complications, or not having presented the disease), inconvenience (self-efficacy, cost, behavioral aspects), lack of confidence (aspects such as distrust in the efficacy and effects adverse effects, psychological aspects related to the link with the authorities and the indications, greater acceptance of negative myths) and calculation (individual and social risk-benefit ratio) [186].

Sociocultural factors were also identified such as economic level, education level (paradoxically, university students reject immunization to a greater extent), religion, and demographics (ethnic differences have been reported in the United States, with a lower immunization rate in Hispanic-Latino populations). Among the countries of the Americas, confidence in vaccination by patients is unevenly distributed. Recent data from the CorCOVID-LATAM study, conducted in 13 Latin American countries, has reported differences in the vaccination rate according to economic income and educational level [187].

In contrast to medical knowledge and conviction, external factors that affect vaccination are grouped together, increasing patients’ hesitancy. Fake news in the media and social networks and people who advocate against vaccination are key aspects in the hesitation process, with potential harmful effects on population health [188]. Years of scientific research can be overshadowed by a simple fake news article developed in one minute and massively disseminated on social media [189].

In an analysis of 450,000 health-related fake news articles collected on social media in Poland, the majority were related to vaccines [190]. Cautious dissemination of recent scientific articles, review of data or publications by experts, social media campaigns and alliances with influential subjects in social networks, as well as public commitment by doctors, are some of the suggestions to overcome this [191,192,193,194,195].

More recently, the COVID-19 pandemic occurred in a completely virtual era with a high global penetrance rate of the internet and social networks, where skepticism about the disease, as well as the safety and efficacy of new vaccines, could influence short-term influenza vaccination rates. Recently, three of the largest platforms (YouTube, Facebook, and Twitter) unified criteria to prevent the spread of vaccine related fake news [196].

IV coverage is a useful indicator to monitor health interventions. Despite a significant improvement of that indicator in Argentina, Brazil, and Mexico, the expected vaccination rates have not been achieved, reflecting the presence of barriers of all kinds.

In the United States, IV was incorporated as a recommendation for at-risk groups by the CDC expert committee more than a decade ago [197]. In Argentina, despite having policies aimed at free vaccination, the vaccination rate was 51.6% in at-risk groups in 2013 [198,199]. At the other extreme, Brazil has reported that the vaccination rate between 2018 and 2020 was 90% to 95% of the target population [200]. In a review carried out by PAHO between 2005 and 2015 in Mexico, Brazil and Argentina, countries with vaccination policies for older adults, the existence of structural barriers in populations with lower socioeconomic and educational levels was established [177].

An assessment carried out in 137 countries reveals that only 51 (37%) have included the influenza vaccine in their list of essential drugs [201,202]. In Latin America and the Caribbean, 30 countries have a list of essential drugs, however in only 11 (36.6%) the influenza vaccine is recognized as essential medicine [201,202,17].

The cardiologist’s knowledge about the benefit of IV as a primary and secondary CV prevention strategy is also considered a barrier in the Americas. Only in some countries is IV included in the national cardiology guidelines. Furthermore, a survey among young cardiologists showed low knowledge regarding vaccinations, and therefore a low rate of prescription [13].

The direct cost of the vaccine is a major barrier. However, PAHO has created a revolving fund to facilitate lower-cost access to both trivalent and quadrivalent vaccines. In this way, 41 countries and territories of Latin America and the Caribbean have a facilitated vaccine acquisition program [203].

**Figure 1:**
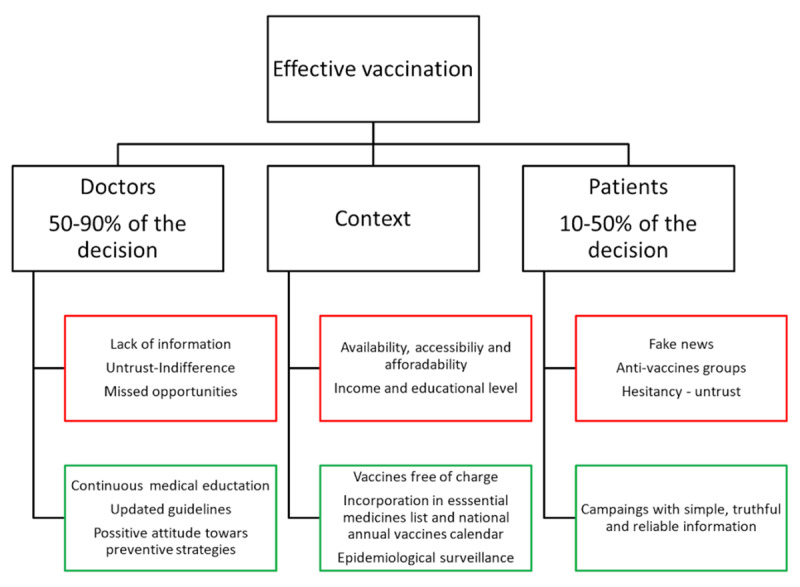
Doctor’s, context, and patient’s role in effective vaccination. Red squares: barriers, green squares: facilitators.

Finally, the CorCOVID LATAM study recently published by IASC demonstrated that there are profound regional differences in vaccination rates [187]: they were very low in the tropical countries of the Americas compared to the countries of the Southern Cone (approximately 50% less), which may partly reflect medical lack of knowledge regarding the circulation of influenza and seasonal peaks even in tropical countries. Of note, that study also found differences in vaccination rates in relation to economic strata and the educational level of the patients. Figure 1 illustrates the main barriers that involve clinicians, patients and their context and the importance of doctor/patient interaction in relation to vaccination rates.

The conclusions related to this issue are summarized in Table 7.

**Table 7:** Barriers to influenza vaccination implementation related to physicians, patients, and their context.

	Conclusions	CoR	LoE

**A**	Medical conviction is the main determinant (50–90%) of effective vaccination. Prescription or advice from a physician or healthcare worker is positively associated with effective vaccination.	I	C-LD
**B**	There are factors beyond access to the vaccine, which psychologically influence the patient’s decision to get vaccinated, encompassed in the concept of hesitancy. Cultural, geographic, economic, religious, and ethnic differences were found as determinants of the vaccination rate.	I	B-NR
**C**	The growing impact of fake news on mass media and social media contributes to the determinants of non-vaccination.	IIa	C-LD

### Effective strategies in increasing influenza vaccination rate

From what has been previously discussed, it is understood that physicians’ conviction seems to be more influential than the perceptions of the patient when analyzing effective vaccination rates. The strong and positive attitude by the doctors at the moment of vaccine prescription seems decisive [177,178,204,205,206,207].

The implementation of continuous medical education programs aimed at *general practitioners* and *specialists* addressing the benefits and opportunities of IV should be considered as a primary objective, as well as its incorporation into clinical practice guidelines.

Immunization should be considered an essential topic of undergraduate medical and nursing schools. The study of immunological aspects, as well as pharmacology, should be expanded and standardized.

The incorporation of advanced medical and nursing students in vaccination campaigns could contribute in this regard.

Patient’s adherence to treatment while undergoing acute-high-mortality diseases such as ACS, is almost complete upon discharge from the coronary care unit, but drastically falls during follow-up [207]. Therefore, implementation of IV prior to discharge or immediately after it would be a highly effective measure to increase vaccination rates.

The access of patients to simple, truthful, and reliable information, carried out through communication campaigns to the community, should also be considered a primary objective. A recent document developed by *Vaccines 4 life* and by the International Federation of Aging [208], has established a framework for the optimal development of vaccination campaigns for influenza.

The recommendations related to this issue are summarized in Table 8.

**Table 8:** Strategies for increasing influenza vaccination rate.

	Recommendations	CoR	LoE

**Physicians**	Develop continuing medical education programs aimed at general practitioners and specialists that address the benefits and opportunities of IV, as well as its incorporation into the clinical practice guidelines. Incorporate the concept of vaccination as a CV prevention strategy together with other preventive interventions. Generate multimodal interventions aimed at outpatient doctors, nurses and students of both careers that allow the dissemination of this concept. Vaccinate prior to or immediately after discharge in patients with acute coronary syndrome. Increase the availability of vaccines in outpatient clinics.	IIa	C-EO
**Patients**	Educate patients and have strategies to overcome vaccine related hesitancy with simple, truthful, and reliable information. Carry out effective vaccination campaigns adapted to local or regional needs. Use clear, simple, multimodal communication oriented at the target population. Refute fake news and promote dialogue with anti-vaccines groups.	IIa	C-EO
**Context**	Improve access to IV, guaranteeing its free provision to target populations. Incorporate IV into the list of essential medicines. Incorporate IV into the annual vaccination calendar. Develop epidemiological surveillance programs to measure results (annual vaccination rates in risk groups).	IIa	C-EO

## Conclusions

There is a strong causal relationship between acute respiratory infections and the incidence of CV events. The severe morbidity and mortality associated with influenza is due in part to the presence of these complications. Incorporating a practice as simple as vaccination can considerably reduce the risk of CV events in selected populations.

With the existing evidence, scientific societies and governmental health agencies strongly recommend the incorporation of IV in patients with pre-existing CV disease and in high-risk groups (people over 65 years of age, HTN, diabetes). Despite this, vaccination rates are far from the expected rates, globally and particularly in the Americas.

The correct understanding of implementation barriers, which involve doctors, patients, and their context, is essential when designing continuous improvement strategies in order to optimize this reality. **The current and unavoidable challenge for our scientific societies is to turn our recommendations into action.**

## Data Accessibility Statement

The data presented in this study is available upon request directed to the corresponding author. An extended version of this document including specific methods of the systematic review, consensus processes, and full tables with results and references; is freely available at www.siacardio.com [17].
